# [Corrigendum] Hypoxia and macrophages promote glioblastoma invasion by the CCL4-CCR5 axis

**DOI:** 10.3892/or.2026.9070

**Published:** 2026-02-04

**Authors:** Ying Wang, Tao Liu, Ning Yang, Shuo Xu, Xingang Li, Donghai Wang

Oncol Rep 36: 3522–3528, 2016; DOI: 10.3892/or.2016.5171

Subsequently to the publication of the above paper, an interested reader drew to the authors' attention that, in comparing Figs. 1A and 2B, which both showed the results of invasion assay experiments, the ‘Normoxia / U87’ data panel in Fig. 1A appeared to overlap with the ‘CCR5 siRNA’ panel in Fig. 2B, whereas the ‘Hypoxia / U87-Mφ’ panel in Fig. 1A also appeared to overlap with the ‘Control siRNA’ panel in Fig. 2B [also note that an expression of concern statement (doi.org/10.3892/or.2025.8989) was issued for this paper].

The authors were able to re-examine their original data files, and realized that the images for Fig. 2B had been inadverently selected incorrectly. The revised version of Fig. 2, containing the correct data for the invasion assay experiments in Fig. 2B, is shown on the next page. Note that the corrections made to this figure do not affect the overall conclusions reported in the paper. The authors are grateful to the Editor of *Oncology Reports* for allowing them the opportunity to publish this Corrigendum, and apologize to the readership for any inconvenience caused.

## Figures and Tables

**Figure 2.**
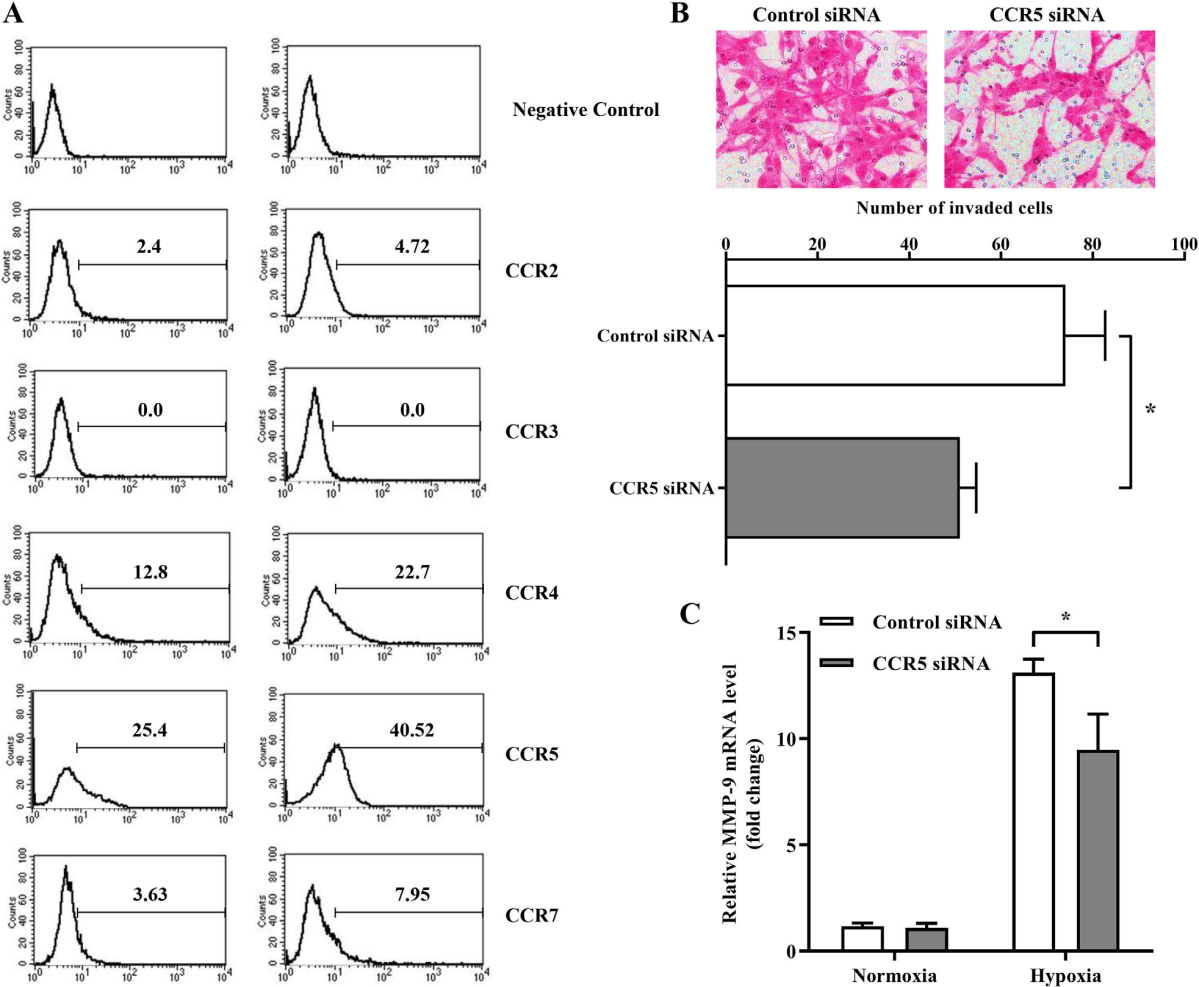
Hypoxia promotes MMP-9 expression and invasion of U87 cells by upregulating CCR5. (A) The expression of CCR2, CCR3, CCR4, CCR5 and CCR7 on U87 cells was determined by flow cytometry. (B) The number of invasive U87 cells in hypoxia with/without CCR5 siRNA transfection. Number of invasive cells was the mean value of five random fields under a light microscope. Original magnification, ×400. (C) The transcription of MMP-9 under the treatment of normoxia or hypoxia with/without CCR5 siRNA transfection. The RT-PCR data were normalized to the control and shown as the fold-change. Each bar represents the mean ± SD (n=3, *p<0.05).

